# Poly[[diaqua-μ_3_-citrato-praseodymium(III)] monohydrate]

**DOI:** 10.1107/S1600536811023804

**Published:** 2011-06-25

**Authors:** Li-Jun Han, Yuan-Fu Deng, Seik Weng Ng

**Affiliations:** a School of Chemistry and Chemical Engineering, South China University of Technology, Guangzhou 510640, People’s Republic of China; b Department of Chemistry, University of Malaya, 50603 Kuala Lumpur, Malaysia

## Abstract

In the coordination polymer, {[Pr(C_6_H_5_O_7_)(H_2_O)_2_]·H_2_O}_*n*_, seven of the nine coordination sites of the monocapped square-anti­prismatic geometry are occupied by three O atoms of the same citrate trianion (an O atom of the hy­droxy unit and the formally single-bond O atoms from two carboxyl units). Two other coordination sites are occupied by the O atoms of a chelating carboxyl unit of another citrate; one of these atoms is additionally involved in bridging. The seventh coordination site is occupied by the O atom of the formally double-bond O atom of a neighboring citrate. The remaining two coordination sites are occupied by water mol­ecules. The citrate functions in a μ_3_-bridging mode, connecting the metal atoms into a ribbon structure parallel to [010]. The structure is consolidated into a three-dimensional network by O—H⋯O hydrogen bonds.

## Related literature

For isotypic [Eu(C_6_H_5_O_7_)(H_2_O)_2_]·H_2_O, see: Tang *et al.* (2011 ▶). 
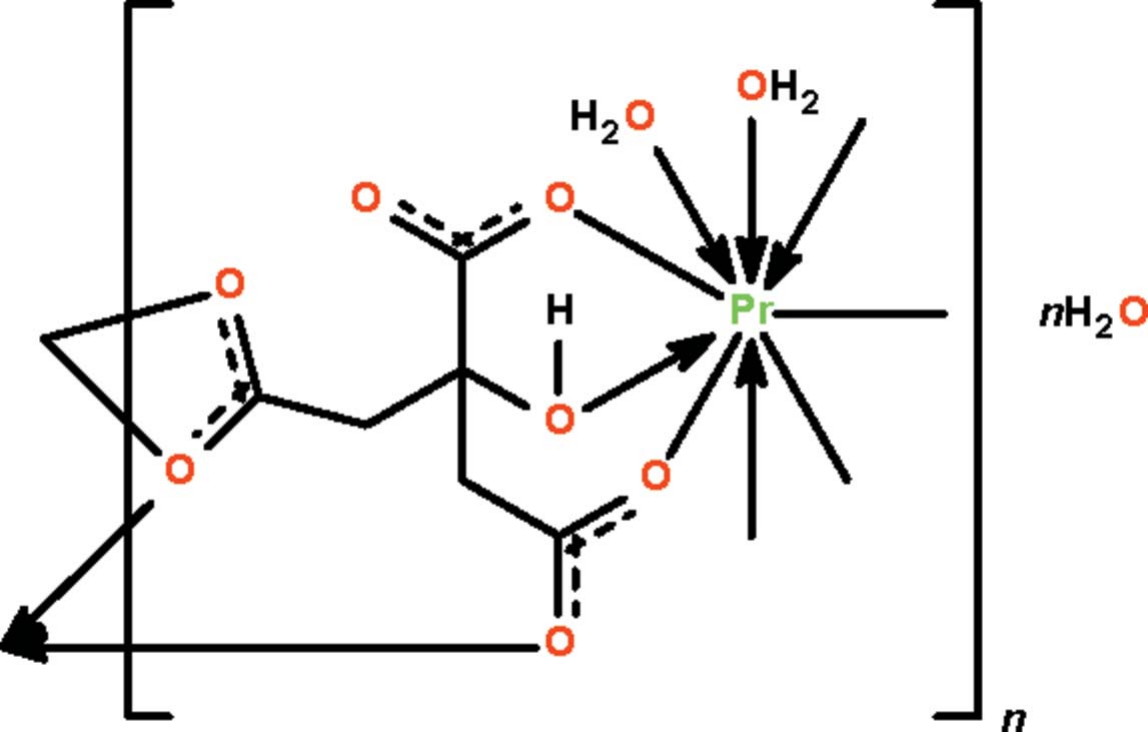


## Experimental

### 

#### Crystal data

[Pr(C_6_H_5_O_7_)(H_2_O)_2_]·H_2_O
*M* *_r_* = 384.06
Monoclinic, 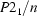

*a* = 6.2645 (3) Å
*b* = 9.7356 (7) Å
*c* = 17.0425 (10) Å
β = 91.0672 (18)°
*V* = 1039.22 (11) Å^3^
*Z* = 4
Mo *K*α radiation
μ = 4.74 mm^−1^
*T* = 293 K
0.30 × 0.15 × 0.10 mm

#### Data collection

Rigaku R-AXIS RAPID diffractometer
Absorption correction: multi-scan (*ABSCOR*; Higashi, 1995 ▶) *T*_min_ = 0.331, *T*_max_ = 0.649
9596 measured reflections
2366 independent reflections
2182 reflections with *I* > 2σ(*I*)
*R*_int_ = 0.038

#### Refinement

*R*[*F* ^2^ > 2σ(*F* ^2^)] = 0.026
*wR*(*F* ^2^) = 0.050
*S* = 1.18
2366 reflections
175 parameters
10 restraints
H atoms treated by a mixture of independent and constrained refinement
Δρ_max_ = 0.76 e Å^−3^
Δρ_min_ = −0.81 e Å^−3^

### 

Data collection: *RAPID-AUTO* (Rigaku, 1998 ▶); cell refinement: *RAPID-AUTO*; data reduction: *CrystalStructure* (Rigaku/MSC, 2002 ▶); program(s) used to solve structure: *SHELXS97* (Sheldrick, 2008 ▶); program(s) used to refine structure: *SHELXL97* (Sheldrick, 2008 ▶); molecular graphics: *X-SEED* (Barbour, 2001 ▶); software used to prepare material for publication: *publCIF* (Westrip, 2010 ▶).

## Supplementary Material

Crystal structure: contains datablock(s) global, I. DOI: 10.1107/S1600536811023804/si2362sup1.cif

Structure factors: contains datablock(s) I. DOI: 10.1107/S1600536811023804/si2362Isup2.hkl

Additional supplementary materials:  crystallographic information; 3D view; checkCIF report

## Figures and Tables

**Table 1:** Hydrogen-bond geometry (Å, °)

*D*—H⋯*A*	*D*—H	H⋯*A*	*D*⋯*A*	*D*—H⋯*A*
O7—H7⋯O2^i^	0.83 (1)	1.72 (1)	2.536 (3)	167 (4)
O1w—H11⋯O2^ii^	0.84 (1)	1.84 (1)	2.666 (3)	169 (4)
O1w—H12⋯O3^iii^	0.84 (1)	1.89 (2)	2.692 (3)	159 (3)
O2w—H21⋯O1w^iv^	0.84 (1)	2.09 (2)	2.854 (4)	151 (4)
O2w—H22⋯O3w	0.84 (1)	1.89 (1)	2.718 (4)	168 (4)
O3w—H31⋯O6^v^	0.84 (1)	2.05 (2)	2.856 (4)	160 (6)

Symmetry codes: (i) 
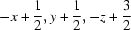
; (ii) 
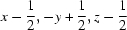
; (iii) 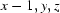; (iv) 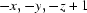; (v) 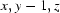.

**Fig. 1.**
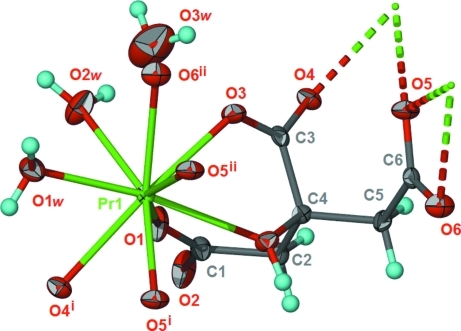
Thermal ellipsoid plot (Barbour, 2001) of a portion of polymeric Pr(H2O)2(C6H5O7).H2O with the 70% probability level; hydrogen atoms are drawn as spheres of arbitrary radius.

**Fig. 2.**
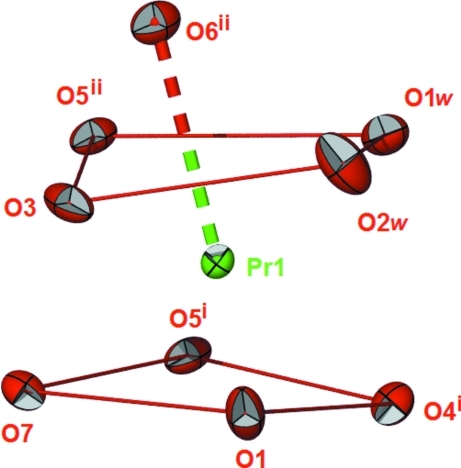
Nine-coordinate geometry of PrIII.

## supplementary crystallographic information

### Comment

A recent report describes the synthesis of Eu(H_2_O)_2_(C_6_H_5_O_7_)^.^H_2_O, a citrate(3-) based coordination polymer that exhibits useful luminescence; the ribbon motif propagates along the *a*-axis and adjacent chains are linked by O–H···O hydrogen bonds into a three-dimensional network. The presence of manganese dichloride is crucial to the synthesis (Tang *et al.*, 2011). The present Pr analog (Scheme I) is isostructural, the two compounds crystallizing with matching cell dimensions. In the coordination polymer, Pr(H_2_O)_2_(C_6_H_5_O_7_)^.^H_2_O (Fig. 1), seven of the nine coordination sites a mono-capped square-antiprismatic geometry (Fig. 2) are occupied by three O atoms of the same citrate trianion (an O atom of the hydroxy unit and the formally single-bond O atoms from two carboxyl units). Two other coordination sites are occupied by the O atoms of a chelating carboxyl unit of another citrate; one of these atoms is additionally involved in bridging. The seventh coordination site is occupied by the O atom of the formally double-bond O atom of a neighboring citrate. The remaining two coordination sites of the are occupied by water molecules. The citrate functions in a *µ*_3_– bridging mode to connect the metal atoms into a ribbon structure. The structure is consolidated into a three-dimensional network by O–H···O hydrogen bonds (Table 1).

### Experimental

Praseodymium oxide, Pr_6_O_11_ (0.341 g), was suspended in water (20 ml) and to the suspension was added manganese dichloride tetrahydrate (0.395 g, 2.0 mmol) and citric acid monohydrate (0.841 g, 4.0 mmol). The mixture was placed in a 25 ml, teflon-lined, stainless-steel Parr bomb. The bomb was heated at 393 K for 72 h. It was cooled to room temperature at 30 K an hour. Green crystals were isolated in 75% yield based on Pr_6_O_11_.

### Refinement

Carbon-bound H atoms treated as riding (C–H 0.97 Å) and their temperature factors were tied by a factor of 1.2 times. The hydroxy and water H atoms were located in a difference Fourier map, and were refined with distance restraints of O–H 0.84±0.01 Å and H···H 1.37±0.01 Å. Their temperature factors were tied by a factor of 1.5 times. The (5 6 3), (-6 6 1), (1 9 2), (4 10 2) and (6 7 3) reflections were omitted owing to bad disagreement.

### Crystal data

**Table:**

[Pr(C_6_H_5_O_7_)(H_2_O)_2_]·H_2_O	*F*(000) = 744
*M**_r_* = 384.06	*D*_x_ = 2.455 Mg m^−^^3^
Monoclinic, *P*2_1_/*n*	Mo *K*α radiation, λ = 0.71073 Å
Hall symbol: -P 2yn	Cell parameters from 8369 reflections
*a* = 6.2645 (3) Å	θ = 3.2–27.4°
*b* = 9.7356 (7) Å	µ = 4.74 mm^−^^1^
*c* = 17.0425 (10) Å	*T* = 293 K
β = 91.0672 (18)°	Prism, light green
*V* = 1039.22 (11) Å^3^	0.30 × 0.15 × 0.10 mm
*Z* = 4	

### Data collection

**Table:**

Rigaku R-AXIS RAPID diffractometer	2366 independent reflections
Radiation source: fine-focus sealed tube	2182 reflections with *I* > 2σ(*I*)
graphite	*R*_int_ = 0.038
Detector resolution: 10.000 pixels mm^-1^	θ_max_ = 27.4°, θ_min_ = 3.2°
ω scans	*h* = −8→8
Absorption correction: multi-scan (*ABSCOR*; Higashi, 1995)	*k* = −12→10
*T*_min_ = 0.331, *T*_max_ = 0.649	*l* = −22→22
9596 measured reflections	

### Refinement

**Table:**

Refinement on *F*^2^	Primary atom site location: structure-invariant direct methods
Least-squares matrix: full	Secondary atom site location: difference Fourier map
*R*[*F*^2^ > 2σ(*F*^2^)] = 0.026	Hydrogen site location: inferred from neighbouring sites
*wR*(*F*^2^) = 0.050	H atoms treated by a mixture of independent and constrained refinement
*S* = 1.18	*w* = 1/[σ^2^(*F*_o_^2^) + (0.*P*)^2^ + 1.4664*P*] where *P* = (*F*_o_^2^ + 2*F*_c_^2^)/3
2366 reflections	(Δ/σ)_max_ = 0.001
175 parameters	Δρ_max_ = 0.76 e Å^−^^3^
10 restraints	Δρ_min_ = −0.81 e Å^−^^3^

### Fractional atomic coordinates and isotropic or equivalent isotropic displacement parameters (Å^2^)

**Table:**

	*x*	*y*	*z*	*U*_iso_*/*U*_eq_	
Pr1	0.11415 (2)	0.319324 (16)	0.566983 (9)	0.01012 (6)	
O1	0.2224 (4)	0.2466 (3)	0.70094 (14)	0.0212 (5)	
O2	0.3108 (4)	0.2315 (3)	0.82649 (14)	0.0245 (5)	
O3	0.5035 (3)	0.2913 (2)	0.56851 (14)	0.0195 (5)	
O4	0.7923 (3)	0.2852 (2)	0.64591 (13)	0.0150 (4)	
O5	0.8717 (3)	0.5316 (2)	0.55742 (13)	0.0166 (4)	
O6	0.7309 (4)	0.7354 (2)	0.57242 (13)	0.0185 (5)	
O7	0.3381 (3)	0.5013 (2)	0.63152 (13)	0.0138 (4)	
H7	0.297 (6)	0.574 (2)	0.652 (2)	0.021*	
O1W	−0.1683 (3)	0.2119 (3)	0.47949 (14)	0.0192 (5)	
H11	−0.172 (5)	0.219 (4)	0.4305 (6)	0.029*	
H12	−0.289 (3)	0.230 (4)	0.4971 (18)	0.029*	
O2W	0.1834 (4)	0.0691 (3)	0.56804 (16)	0.0277 (6)	
H21	0.147 (6)	−0.001 (3)	0.543 (2)	0.042*	
H22	0.300 (4)	0.054 (4)	0.591 (2)	0.042*	
O3W	0.5632 (6)	−0.0132 (4)	0.6318 (2)	0.0564 (10)	
H31	0.617 (8)	−0.092 (2)	0.626 (4)	0.085*	
H32	0.635 (8)	0.042 (4)	0.605 (3)	0.085*	
C1	0.3229 (5)	0.2872 (3)	0.76032 (18)	0.0146 (6)	
C2	0.4742 (5)	0.4089 (3)	0.75574 (17)	0.0141 (6)	
H2A	0.6066	0.3849	0.7829	0.017*	
H2B	0.4118	0.4855	0.7836	0.017*	
C3	0.6141 (5)	0.3355 (3)	0.62538 (18)	0.0125 (6)	
C4	0.5269 (4)	0.4564 (3)	0.67268 (17)	0.0109 (6)	
C5	0.6919 (5)	0.5734 (3)	0.67823 (17)	0.0130 (6)	
H5A	0.6285	0.6513	0.7046	0.016*	
H5B	0.8134	0.5429	0.7097	0.016*	
C6	0.7682 (4)	0.6185 (3)	0.59874 (17)	0.0120 (6)	

### Atomic displacement parameters (Å^2^)

**Table:**

	*U*^11^	*U*^22^	*U*^33^	*U*^12^	*U*^13^	*U*^23^
Pr1	0.00959 (9)	0.01080 (10)	0.00997 (9)	0.00001 (6)	0.00066 (6)	0.00069 (6)
O1	0.0237 (12)	0.0235 (13)	0.0161 (12)	−0.0102 (10)	−0.0060 (9)	0.0049 (9)
O2	0.0360 (13)	0.0230 (13)	0.0142 (12)	−0.0143 (11)	−0.0037 (10)	0.0078 (10)
O3	0.0124 (11)	0.0235 (13)	0.0226 (13)	0.0015 (9)	−0.0010 (9)	−0.0123 (10)
O4	0.0116 (10)	0.0164 (11)	0.0171 (11)	0.0030 (9)	0.0016 (8)	0.0017 (9)
O5	0.0217 (11)	0.0133 (11)	0.0149 (11)	0.0037 (9)	0.0072 (8)	0.0010 (9)
O6	0.0234 (12)	0.0155 (12)	0.0167 (12)	0.0033 (9)	0.0039 (9)	0.0031 (9)
O7	0.0144 (10)	0.0114 (11)	0.0157 (11)	0.0035 (8)	−0.0022 (8)	−0.0017 (8)
O1W	0.0158 (11)	0.0267 (13)	0.0151 (12)	−0.0010 (10)	0.0013 (8)	−0.0025 (10)
O2W	0.0334 (14)	0.0158 (13)	0.0336 (15)	0.0004 (11)	−0.0102 (11)	−0.0021 (11)
O3W	0.056 (2)	0.0331 (18)	0.079 (3)	0.0182 (16)	−0.0212 (18)	−0.0082 (18)
C1	0.0145 (14)	0.0156 (16)	0.0138 (15)	−0.0021 (12)	0.0009 (11)	0.0016 (12)
C2	0.0154 (14)	0.0160 (16)	0.0110 (14)	−0.0034 (12)	0.0018 (11)	−0.0004 (12)
C3	0.0107 (14)	0.0129 (15)	0.0141 (15)	−0.0025 (11)	0.0045 (11)	0.0029 (11)
C4	0.0116 (13)	0.0104 (14)	0.0105 (14)	0.0000 (11)	0.0001 (10)	−0.0005 (11)
C5	0.0155 (14)	0.0117 (15)	0.0118 (14)	−0.0040 (12)	0.0022 (10)	−0.0006 (11)
C6	0.0092 (13)	0.0133 (16)	0.0134 (14)	−0.0026 (11)	−0.0005 (10)	−0.0009 (11)

### Geometric parameters (Å, °)

**Table:**

Pr1—O1	2.473 (2)	O6—Pr1^ii^	2.637 (2)
Pr1—O3	2.454 (2)	O7—C4	1.432 (3)
Pr1—O4^i^	2.467 (2)	O7—H7	0.834 (10)
Pr1—O5^i^	2.568 (2)	O1W—H11	0.84 (1)
Pr1—O5^ii^	2.572 (2)	O1W—H12	0.84 (1)
Pr1—O6^ii^	2.637 (2)	O2W—H21	0.84 (1)
Pr1—O7	2.502 (2)	O2W—H22	0.84 (1)
Pr1—O1W	2.520 (2)	O3W—H31	0.84 (1)
Pr1—O2W	2.474 (3)	O3W—H32	0.85 (1)
O1—C1	1.246 (4)	C1—C2	1.520 (4)
O2—C1	1.255 (4)	C2—C4	1.531 (4)
O3—C3	1.257 (4)	C2—H2A	0.9700
O4—C3	1.262 (4)	C2—H2B	0.9700
O4—Pr1^iii^	2.467 (2)	C3—C4	1.533 (4)
O5—C6	1.284 (4)	C4—C5	1.540 (4)
O5—Pr1^iii^	2.568 (2)	C5—C6	1.510 (4)
O5—Pr1^ii^	2.572 (2)	C5—H5A	0.9700
O6—C6	1.244 (4)	C5—H5B	0.9700
			
O3—Pr1—O4^i^	143.27 (8)	C6—O5—Pr1^ii^	96.06 (18)
O3—Pr1—O1	72.72 (8)	Pr1^iii^—O5—Pr1^ii^	118.48 (8)
O4^i^—Pr1—O1	70.79 (7)	C6—O6—Pr1^ii^	94.03 (18)
O3—Pr1—O2W	73.53 (8)	C4—O7—Pr1	116.75 (17)
O4^i^—Pr1—O2W	90.48 (8)	C4—O7—H7	108 (3)
O1—Pr1—O2W	70.50 (9)	Pr1—O7—H7	128 (3)
O3—Pr1—O7	61.63 (7)	Pr1—O1W—H11	124 (3)
O4^i^—Pr1—O7	108.24 (7)	Pr1—O1W—H12	109 (3)
O1—Pr1—O7	69.83 (7)	H11—O1W—H12	109 (2)
O2W—Pr1—O7	126.67 (8)	Pr1—O2W—H21	139 (3)
O3—Pr1—O1W	130.32 (7)	Pr1—O2W—H22	109 (3)
O4^i^—Pr1—O1W	72.22 (7)	H21—O2W—H22	110 (2)
O1—Pr1—O1W	127.21 (8)	H31—O3W—H32	107 (2)
O2W—Pr1—O1W	73.54 (8)	O1—C1—O2	123.7 (3)
O7—Pr1—O1W	159.37 (8)	O1—C1—C2	120.8 (3)
O3—Pr1—O5^i^	132.58 (7)	O2—C1—C2	115.5 (3)
O4^i^—Pr1—O5^i^	69.79 (7)	C1—C2—C4	115.4 (2)
O1—Pr1—O5^i^	116.15 (8)	C1—C2—H2A	108.4
O2W—Pr1—O5^i^	153.65 (8)	C4—C2—H2A	108.4
O7—Pr1—O5^i^	77.53 (7)	C1—C2—H2B	108.4
O1W—Pr1—O5^i^	83.60 (7)	C4—C2—H2B	108.4
O3—Pr1—O5^ii^	91.25 (8)	H2A—C2—H2B	107.5
O4^i^—Pr1—O5^ii^	124.51 (7)	O3—C3—O4	123.6 (3)
O1—Pr1—O5^ii^	155.43 (7)	O3—C3—C4	118.1 (3)
O2W—Pr1—O5^ii^	123.56 (8)	O4—C3—C4	118.3 (3)
O7—Pr1—O5^ii^	86.28 (7)	O7—C4—C2	110.8 (2)
O1W—Pr1—O5^ii^	77.36 (8)	O7—C4—C3	106.0 (2)
O5^i^—Pr1—O5^ii^	61.52 (8)	C2—C4—C3	109.8 (2)
O3—Pr1—O6^ii^	66.72 (7)	O7—C4—C5	110.6 (2)
O4^i^—Pr1—O6^ii^	141.46 (7)	C2—C4—C5	108.8 (2)
O1—Pr1—O6^ii^	132.42 (8)	C3—C4—C5	110.8 (2)
O2W—Pr1—O6^ii^	74.95 (8)	C6—C5—C4	112.5 (2)
O7—Pr1—O6^ii^	109.04 (7)	C6—C5—H5A	109.1
O1W—Pr1—O6^ii^	69.47 (7)	C4—C5—H5A	109.1
O5^i^—Pr1—O6^ii^	109.42 (7)	C6—C5—H5B	109.1
O5^ii^—Pr1—O6^ii^	49.65 (7)	C4—C5—H5B	109.1
C1—O1—Pr1	141.7 (2)	H5A—C5—H5B	107.8
C3—O3—Pr1	120.28 (19)	O6—C6—O5	119.9 (3)
C3—O4—Pr1^iii^	121.60 (19)	O6—C6—C5	122.0 (3)
C6—O5—Pr1^iii^	143.1 (2)	O5—C6—C5	118.1 (3)
			
O3—Pr1—O1—C1	67.4 (4)	Pr1—O3—C3—O4	152.9 (2)
O4^i^—Pr1—O1—C1	−116.8 (4)	Pr1—O3—C3—C4	−26.6 (4)
O2W—Pr1—O1—C1	145.6 (4)	Pr1^iii^—O4—C3—O3	72.0 (4)
O7—Pr1—O1—C1	2.0 (3)	Pr1^iii^—O4—C3—C4	−108.5 (3)
O1W—Pr1—O1—C1	−164.6 (3)	Pr1—O7—C4—C2	−83.4 (2)
O5^i^—Pr1—O1—C1	−62.1 (4)	Pr1—O7—C4—C3	35.7 (3)
O5^ii^—Pr1—O1—C1	16.1 (5)	Pr1—O7—C4—C5	155.86 (18)
O6^ii^—Pr1—O1—C1	99.8 (4)	C1—C2—C4—O7	61.8 (3)
O4^i^—Pr1—O3—C3	−49.7 (3)	C1—C2—C4—C3	−55.0 (3)
O1—Pr1—O3—C3	−43.1 (2)	C1—C2—C4—C5	−176.4 (3)
O2W—Pr1—O3—C3	−117.3 (2)	O3—C3—C4—O7	−6.7 (4)
O7—Pr1—O3—C3	32.9 (2)	O4—C3—C4—O7	173.7 (2)
O1W—Pr1—O3—C3	−167.6 (2)	O3—C3—C4—C2	113.0 (3)
O5^i^—Pr1—O3—C3	66.8 (3)	O4—C3—C4—C2	−66.5 (3)
O5^ii^—Pr1—O3—C3	118.0 (2)	O3—C3—C4—C5	−126.7 (3)
O6^ii^—Pr1—O3—C3	162.4 (3)	O4—C3—C4—C5	53.7 (4)
O3—Pr1—O7—C4	−35.86 (18)	O7—C4—C5—C6	−63.2 (3)
O4^i^—Pr1—O7—C4	105.51 (18)	C2—C4—C5—C6	175.0 (2)
O1—Pr1—O7—C4	44.86 (18)	C3—C4—C5—C6	54.1 (3)
O2W—Pr1—O7—C4	0.7 (2)	Pr1^ii^—O6—C6—O5	6.0 (3)
O1W—Pr1—O7—C4	−166.6 (2)	Pr1^ii^—O6—C6—C5	−173.0 (2)
O5^i^—Pr1—O7—C4	169.07 (19)	Pr1^iii^—O5—C6—O6	153.9 (2)
O5^ii^—Pr1—O7—C4	−129.30 (19)	Pr1^ii^—O5—C6—O6	−6.2 (3)
O6^ii^—Pr1—O7—C4	−84.44 (19)	Pr1^iii^—O5—C6—C5	−27.1 (4)
Pr1—O1—C1—O2	164.9 (2)	Pr1^ii^—O5—C6—C5	172.8 (2)
Pr1—O1—C1—C2	−16.3 (5)	Pr1^iii^—O5—C6—Pr1^ii^	160.0 (3)
O1—C1—C2—C4	−11.9 (4)	C4—C5—C6—O6	114.9 (3)
O2—C1—C2—C4	167.0 (3)	C4—C5—C6—O5	−64.0 (3)

Symmetry codes: (i) *x*−1, *y*, *z*; (ii) −*x*+1, −*y*+1, −*z*+1; (iii) *x*+1, *y*, *z*.

### Hydrogen-bond geometry (Å, °)

**Table:**

*D*—H···*A*	*D*—H	H···*A*	*D*···*A*	*D*—H···*A*
O7—H7···O2^iv^	0.83 (1)	1.72 (1)	2.536 (3)	167 (4)
O1w—H11···O2^v^	0.84 (1)	1.84 (1)	2.666 (3)	169 (4)
O1w—H12···O3^i^	0.84 (1)	1.89 (2)	2.692 (3)	159 (3)
O2w—H21···O1w^vi^	0.84 (1)	2.09 (2)	2.854 (4)	151 (4)
O2w—H22···O3w	0.84 (1)	1.89 (1)	2.718 (4)	168 (4)
O3w—H31···O6^vii^	0.84 (1)	2.05 (2)	2.856 (4)	160 (6)

Symmetry codes: (iv) −*x*+1/2, *y*+1/2, −*z*+3/2; (v) *x*−1/2, −*y*+1/2, *z*−1/2; (i) *x*−1, *y*, *z*; (vi) −*x*, −*y*, −*z*+1; (vii) *x*, *y*−1, *z*.

## References

[bb1] Barbour, L. J. (2001). *J. Supramol. Chem.* **1**, 189–191.

[bb2] Higashi, T. (1995). *ABSCOR* Rigaku Corporation, Tokyo, Japan.

[bb3] Rigaku (1998). *RAPID-AUTO* Rigaku Corporation, Tokyo, Japan.

[bb4] Rigaku/MSC (2002). *CrystalStructure* Rigaku/MSC, The Woodlands, Texas, USA.

[bb5] Sheldrick, G. M. (2008). *Acta Cryst.* A**64**, 112–122. 10.1107/S0108767307043930 18156677

[bb6] Tang, S.-D., Deng, Y.-F. & Zhan, S.-Z. (2011). *Chin. J. Struct. Chem.* **30**, 424–430.

[bb7] Westrip, S. P. (2010). *J. Appl. Cryst.* **43**, 920–925.

